# Anticancer activities of toxic isolate of *Xestospongia testudinaria* sponge

**DOI:** 10.14202/vetworld.2019.1434-1440

**Published:** 2019-09

**Authors:** Made Dira Swantara, Wiwik Susanah Rita, Nyoman Suartha, Kadek Karang Agustina

**Affiliations:** 1 Department of Applied Chemistry, Graduate School, Udayana University, Denpasar, Indonesia; 2 Department of Chemistry, Udayana University, Badung, Indonesia; 3 Department of Internal Medicine, Faculty of Veterinary Medicine, Udayana University, Denpasar, Indonesia; 4 Department of Public Health, Faculty of Veterinary Medicine, Udayana University, Denpasar, Indonesia

**Keywords:** anticancer activity, HeLa cell, *Xestospongia testudinaria*

## Abstract

**Aims:**

The purposes of this study were to determine the anticancer activity of *Xestospongia testudinaria* sponge isolate and identify the responsible compounds.

**Materials and Methods:**

The metabolites were extracted using methanol maceration at room temperature. The separation and purification of metabolites were performed using fractionation and column chromatography. The toxicity was examined using the brine shrimp lethality assay, and the toxic isolates were tested for anticancer activity against HeLa cells. Gas chromatography-mass spectrometry analysis was used to identify the compounds in the isolate.

**Results:**

When the methanol extract was partitioned with n-hexane, chloroform, and n-butanol, the chloroform fraction was the most toxic, with a concentration that results in 50% lethality (LC_50_) value of 39.81 ppm. After separation of the chloroform extract, fraction B (FB) was the most toxic, with an LC_50_ value of 44.67 ppm. The isolate from FB showed anticancer activity with a concentration at which 50% of growth was inhibited (IC_50_) of 2.273 ppm. In total, 21 compounds were identified in anticancer isolates: Nonanedioic acid; tetradecanoic acid; trans-phytol; 2-pentadecanone-6,10,14-trimethyl; pentadecanoic acid; 2-hexadecen-1-ol, 3,7,11,15-tetramethyl-; pentadecanoic acid; 2-hexadecen-1-ol, 3,7,11,15-tetramethyl-; 2,3,7-trimethyloctanal; palmitic acid; docosanoic acid, ethyl ester; 1,E-11,Z-13-octadecatriene; chloromethyl 4-chlorododecanoate; 1-tricosene; 9,12-octadecadienoic acid; 4,8,12,16-tetramethylheptadecan-4-olide; 1-docosene; heneicosane; phosphonic acid, dioctadecyl ester; dodecane,4,6-dimethyl-; n-tetratriacontane; 1-iodohexadecane; and n-heneicosane.

**Conclusion:**

These findings indicate that the isolate of *X. testudinaria* can be used as a natural anticancer toward HeLa cell.

## Introduction

Cancer is caused by the abnormal growth and development of cells in the body [1]. During the course of the disease, these cells may spread to other parts of the body, ultimately resulting in death where the currently available therapies are not effective [2-5]. Cancer is the second most common cause of death after heart disease, responsible for more than 500,000 deaths per year in the United States [6] and an estimated 100 new patients in every 100,000 inhabitants diagnosed every year in Indonesia [7]. Current cancer treatments generally combine surgical and radiation methods with chemotherapy [8], but the methods have not led to optimal results.

The development of new anticancer drugs has become a priority because of the high cost and low selectivity of the currently available drugs [9]. A wide range of natural resources, including marine organisms, has been considered in the search for new anticancer compounds [10]. Some researchers believe that sponges are a potential source of bioactive compounds from the ocean. Nearly 5000 compounds have been isolated from these sponges and determined to have various biological effects such as antimicrobial, antifungal, antiviral, and anticancer activities [11]. Metabolite extracts from sea sponges contain bioactive compounds with antiviral [12], anti-HIV, anti-inflammatory, antifungal, antileukemia [13], enzyme inhibitory [14], antimalarial [15], antioxidant [16], cytoprotective, and antitumor [17] activity.

Few studies have assessed the anticancer activity of *Xestospongia testudinaria* sponge. The alcohol and n-hexane extracts (EH) from *X. testudinaria* from the Saudi Red Sea were found to have strong cytotoxic activity against human cervical cancer (HeLa), human hepatocellular carcinoma (HepG-2), and human medulloblastoma (Daoy) cell lines [18]. The methanol extract of *X. testudinaria* sponge from Sanur, Bali, Indonesia had anticancer activity against HeLa cells with an IC_50_ value of 1327 ppm [19]. The antitumor activity of two new polyacetylene sponges *Xestospongia* spp. from the Red Sea was also reported [20]. Moreover, effects other than anticancer of *X. testudinaria* sponge extract have been widely reported: *X. testudinaria* sponge showed antibacterial activity [20-22]; Vietnamese *X. testudinaria* sponge has antifouling activity of 26,27-cyclosterols [23]; the toxicity of the methanol extract of *X. testudinaria* sponge was reported to have a concentration that results in 50% lethality (LC_50_) value of 31.62 ppm [24,25]. Five compounds identified in the sponges (sapinofuranone, xestospongic acid, 24-hydroperoxy-24-vinyl-cholesterol, saringosterol, and 29-hydroperoxystigmasta-5,24-dien-3β-ol) were toxic to *Artemia salina* larvae with LC_50_ values between 0.56 and 6.99 μM [24].

A preliminary test for anticancer activity was conducted using the brine shrimp lethality assay [26]. If material has an LC_50_ value of below 1000 ppm, this indicates anticancer potential and suggests the use of further anticancer testing in HeLa cells [19,27]. The methanol extract of *X. testudinaria* sponge from Sanur Bali had anticancer activity with a concentration at which 50% of growth was inhibited (IC_50_) of 1327 ppm [19].

The purposes of this study were to determine the anticancer activities of toxic isolates of *X. testudinaria* sponge from Sanur, Bali, Indonesia, and identify the responsible compounds.

## Materials and Methods

### Ethical approval

The study only used invertebrate, so ethical approval was not necessary.

### Materials

The *X. testudinaria* sponge was collected from the coastal waters of Sanur, Bali, on May 9, 2018. Methanol, n-hexane, chloroform, and n-butanol were purchased from Merck, Germany. Brine shrimp *Artemia salina* eggs were purchased from American Technology. The cell line was purchased from the Primate Study Centre, Bogor Agriculture University. Gas chromatography-mass spectrometry (GC-MS) was performed using a GC-MS-QP2010 Ultra Shimadzu from Japan.

### Sample preparation and extraction

Fresh sponge samples were washed with water until clean, cut into small pieces, and dried away from direct sunlight for 7 days. After drying, the sample was sieved through at 100 mesh filter to ensure appropriate homogeneity. In total, 500 g dry powder of sample was extracted using methanol maceration. Every 24 h, the extract was filtered, and the pulp was re-extracted using fresh methanol. This extraction process was conducted 3 times. All the methanol extracts were evaporated using a rotary evaporator to yield the crude extract [19].

### Fractionation

The crude extract (10 g) was completely dissolved in a methanol-water mixture (3:7), and then the methanol was removed by evaporation. The water extract was fractioned successively with n-hexane (3×100 mL), chloroform (3×100 mL), and n-butanol (3×100 mL). The solvents were removed by evaporation to obtain the EH, chloroform extract (EC), and n-butanol extract (EB). All three extracts (EH, EC, and EB) were tested for toxicity [19].

### Separation and purification

The most toxic extracts were then separated by silica gel column chromatography using suitable eluents to obtain several fractions. All fractions were tested for toxicity. The most toxic fraction was tested for purity by thin-layer chromatography (TLC) using several eluent systems. If the isolate provided a single stain on the TLC plate in various eluent systems, then the isolate was considered pure according to TLC; finally, the anticancer effect of the isolate was determined in HeLa cells [19].

### Toxicity test

The medium for larvae hatching was made by filtering seawater. Seawater was placed in an aquarium, which was divided into two parts; one was dark and the other was bright. *A. salina* eggs (50 mg) were placed or immersed in the dark part and left for 48 h until it hatched into a mature larva and was ready to use for testing. Each methanol and n-EH (20 mg) were dissolved into 2 mL of solvent. These solutions were considered as 500 μL, 50 μL, and 5 μL, respectively; each solution was inserted into the test tube, and the solvent was evaporated. Dimethyl sulfoxide (nearly 50 μL), seawater (1 mL), and 10 larvae were placed into a test tube containing the sample; the solvent was evaporated, and seawater was added to a volume of 5 mL, to obtain extract concentrations of 1000, 100, and 10 ppm. A concentration of 0 ppm (solution without the addition of the extract) was prepared as a control. After 24 h, the death of *A. saline* larvae was measured. The standard assessment of larval mortality is when the larvae do not show movement during an observation period of several seconds [28]. The number of live and dead larvae was recorded, and the data were analyzed to find the LC_50_.

### Anticancer test

The toxic isolate was assayed for its anticancer activity against HeLa cells [29]. HeLa cells were cultured in Roswell Park Memorial Institute 1640 medium, and the initial number of cells was counted using a microscope. The cells were trypsinized, harvested, and centrifuged to form two layers (sediment and supernatant). The supernatant was removed and the precipitate was pelletized; 1 mL of complete medium was added and then the number of cells as counted using a hemocytometer. Subsequently, 2×10^4^ cells were seeded in 100 μL of the medium in a 96-well plate and incubated for 1–2 h to allow the cells to adhere. Subsequently, 100 µL extracts of the test material were added at various concentrations (1000, 500, 250, 125, 62.5, 31.25, 15.62, 7.81, 3.91, 1.95, 0.97, 0.48, 0.24, 0.12, and 0.06 μg/mL), to make a total volume of 200 µL in each well. The cells were then incubated for 24 h at 37°C. After 24 h, the cells were observed using the microscope. 3-(4,5-Dimethylthiazole-2-yl)-2,5-diphenyltetrazolium bromide (MTT; 5 μg/mL) was added to each and incubated for 4 h. Subsequently, stop solution of sodium dodecyl sulfate (SDS) 10% in 0.01 N HCl was added into each well and incubated overnight. The absorbance at 500 nm was observed using an enzyme-linked immunosorbent assay plate reader.

### Identification of compounds

Anticancer compounds were separated using GC-MS. The mass spectrum of the active isolates obtained was compared with standard reference spectra that were programmed on the device (GC-MS-QP2010 Ultra Shimadzu) [30,31].

### Anticancer activity

Anticancer activity against HeLa cells was determined using the MTT assay. The MTT assay is a colorimetric method used to measure cell proliferation. The principle of the assay is the reduction of yellow tetrazolium salt MTT, which is reduced to purple formazan crystals by living mitochondria [32]: MTT is absorbed into live cells and reduced by succinate dehydrogenase in the electron transport chain of mitochondria to formazan. The formed formazan crystals are dissolved in 10% SDS, forming a purple solution. The MTT reduction occurs through the NADH and NADPH cofactor pyridine nucleotides, which only occur in living cells; thus, the amount of formazan formed is proportional to the number of live cells [33]. The optical density (OD) of each well was measured at 595 nm using a microplate reader. All tests were conducted in triplicate, and the average OD value was converted to a percentage inhibition.

## Results

### Sample preparation and extraction

Fresh sample (10 kg) was washed, cut, and dried for 7 days, which produced 953 g of dried samples. The dried samples were refined to produce 781 g of dry sample powder. Subsequently, 500 g of dry sample powder was extracted using methanol, which yielded 67 g of the methanol extract.

### Fractionation

The methanol extract (50 g) was partitioned successively with n-hexane, chloroform, and n-butanol to produce the n-hexane, chloroform, and n-EB, respectively, with yields of 5.17, 3.84, and 9.27 g, respectively. The results of the toxicity test of the three extracts are shown in Table-1. The EC was the most toxic, with an LC_50_ value of 39.81 ppm.

**Table 1:** Toxicity of n-hexane, chloroform, and n-butanol extracts.

Sample	Concentration (ppm)	Number of dead larvae			Mortality (%)	LC_50_ (ppm)

		I	II	III		
EH	0	0	0	0	0	70.79
	10	1	1	2	7	
	100	5	6	6	58	
	1000	10	9	9	94	
EC	0	0	0	0	0	39.81
	10	2	1	1	8	
	100	7	8	8	75	
	1000	10	9	10	95	
EB	0	0	0	0	0	63.09
	10	2	2	1	13	
	100	5	6	7	58	
	1000	10	9	10	100	

EH=Hexane extract, EC=Chloroform extract, EB=N-butanol extract

### Separation and purification

The EC was separated by silica gel column chromatography using n-hexane-chloroform (1.5:8.5) as an eluent, which produced four fractions (Fraction A, Fraction B [FB], Fraction C, and Fraction D). The toxicity data of the four fractions are shown in Table-2.

**Table 2:** The toxicity of the fraction results from column chromatography.

Sample	Concentration (ppm)	Number of dead larvae			Mortality (%)	LC_50_ (ppm)

		I	II	III		
FA	0	0	0	0	0	50.12
	10	2	1	1	7	
	100	7	7	6	67	
	1000	10	9	9	94	
FB	0	0	0	0	0	44.67
	10	2	1	1	8	
	100	7	7	6	73	
	1000	10	10	9	100	
FC	0	0	0	0	0	56.23
	10	2	2	1	13	
	100	7	6	6	62	
	1000	10	9	9	94	
FD	0	0	0	0	0	79.43
	10	2	2	1	13	
	100	5	5	6	54	
	1000	10	9	9	94	

FA=Fraction A, FB=Fraction B, FC=Fraction C, FD=Fraction D

As shown in Table-2, the most toxic fraction was FB, which had an LC_50_ value of 44.67 ppm. The FB isolate was then tested for purity using the silica gel TLC method. The tests with various eluent systems all resulted in a single stain, which indicated that the FB isolate was pure, according to TLC.

### Anticancer activity

The OD data and percentage inhibition of HeLa cells after treatment with sponge *X. testudinaria* toxic isolate (FB) are presented in Table-3.

**Table 3:** Toxic isolate (FB) inhibition.

Sample (ppm)	OD			Average	Inhibition (%)

	1	2	3		
100	0.046	0.045	0.25	0.038	80.8^a^
50	0.052	0.046	0.032	0.043	78.28^b^
25	0.062	0.056	0.03	0.049	75.25^c^
12.5	0.071	0.056	0.04	0.056	71.71^d^
6.25	0.075	0.063	0.07	0.069	65.15^e^
3.125	0.098	0.083	0.09	0.090	54.54^f^
1.56	0.102	0.1	0.1	0.100	49.49^g^
0.78	0.125	0.115	0.117	0.119	39.89^h^
0.39	0.139	0.140	0.150	0.143	27.77^i^
0.195	0.145	0.155	0.160	0.153	22.72^j^
Cell control	0.195	0.197	0.202	0.198	0.00^k^

*Values followed by the same letters in the same column are not significantly different according to the Duncan’s Multiple Range Test at p<5%. FB = Fraction B, OD = Optical density

**Figure-1:**
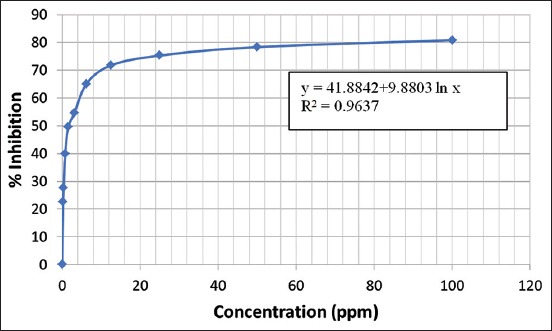
The curve correlation between sample concentration and inhibition.

Based on the data in Table-3, the relationship between sample concentration and inhibition was used to determine the IC_50_ (Figure-1).

The equation of the graph in Figure-1 is y = 47.4729+8.9399ln(x), and the coefficient determination (R^2^) was 0.9393. Therefore, the IC_50_ was calculated from the following equation:

50 = 41.8842+9.8803ln(x)

ln(x) = (50-41.8842)/9.8803 = 0.821412

x = 2.273

The IC_50_ of the toxic isolate from FB was 2.273 ppm, which is considered a very strong activity [34].

### Identification of compounds

**Figure-2:**
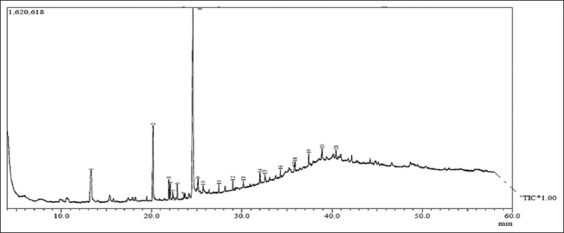
Gas chromatogram of the anticancer isolate of *Xestospongia testudinaria* sponge.

The GC analysis of the anticancer is presented in Figure-2. Twenty-one compounds were present in the isolate. The compounds were identified by MS and are presented in Table-4.

**Table 4:** Compounds identified in the anticancer isolate of *X. testudinaria* sponge.

Peak	Retention time (min)	Abundance (%)	Molecular weight	Molecular formula	Compound name
1	13.283	13.66	188	C_9_H_16_O4	Nonanedioic acid
2	20.167	15.37	228	C_14_H_28_O_2_	Tetradecanoic acid
3	21.917	2.77	296	C_20_H_40_O	Trans-phytol
4	22.067	2.22	268	C_18_H_36_O	2-Pentadecanone-6,10,14-trimethyl
5	22.350	0.99	242	C_15_H_30_O_2_	Pentadecanoic acid
6	22.850	2.02	278	C_20_H_38_	2-Hexadecen-1-ol, 3,7,11,15-tetramethyl-
7	23.575	0.43	170	C_11_H_22_O	2,3,7-Trimethyloctanal
8	24.583	50.24	256	C16H_32_O_2_	Palmitic acid
9	25.150	1.50	368	C_24_H_48_O_2_	Docosanoic acid, ethyl ester
10	25.717	0.75	248	C_18_H_32_	1, E-11, Z-13-octadecatriene
11	27.458	0.88	282	C_13_H_24_Cl_2_O_2_	Chloromethyl 4-chlorododecanoate
12	29.017	1.04	322	C_23_H_46_	1-Tricosene
13	30.183	0.48	294	C_19_H_34_O_2_	9,12-Octadecadienoic acid
14	32.000	1.39	324	C_21_H_40_O_2_	4,8,12,16-Tetramethylheptadecan-4-olide
15	32.550	0.57	308	C_22_H_44_	1-Docosene
16	34.292	0.72	296	C_21_H_44_	Heneicosane
17	35.817	0.69	586	C_36_H_75_O_3_P	Phosphonic acid, dioctadecyl ester
18	35.892	1.12	198	C_14_H_30_	Dodecane, 4,6-dimethyl-
19	37.417	1.52	478	C_34_H_70_	n-Tetratriacontane
20	38.900	0.87	352	C_16_H_33_I	1-Iodohexadecane
21	40.450	0.79	296	C_21_H_44_	n-Heneicosane

*X. testudinaria*=*Xestospongia testudinaria*

## Discussion

The data presented in Tables-1 and 2 show that the cytotoxic potency of *X. testudinaria* sponge extract increased from the EC (39.81 ppm) to FB (44.67 ppm). This indicated that the toxic compounds in the EC exerted a synergistic effect [35]. Based on the anticancer preliminary test (toxicity test), the EC was the most toxic extract, indicating that the toxic compounds are semipolar because they dissolve into chloroform (Table-2) [36]. As shown in Figure-1, the sample concentration was positively correlated with the inhibition of HeLa cell growth. All inhibition percentages of the samples and concentrations were significantly different from the control. Compared with a previous report [19], the anticancer activity of toxic isolates was lower, with an IC_50_ of 2273 ppm. This showed that the anticancer compounds in the *X. testudinaria* sponge exerted synergistic effects.

The anticancer activity (IC_50_ = 2.273 ppm) of *X. testudinaria* sponge toxic isolate agreed with to a previous report [18], in which the IC_50_ of ethanol extract of *X. testudinaria* sponge on HeLa, HepG-2, and Daoy cells was 83.35, 23.45, and 23.31 ppm, respectively. In addition, the n-EH from the same sponge had IC_50_ values of 33.7, 30.2, and 20.74 ppm in of on HeLa, HepG-2, and Daoy cells, respectively.

We found some compounds of anticancer isolates in Table-4 were fatty acids and esters (nonanedioic acid, tetradecanoic acid, pentadecanoic acid, palmitic acid, docosanoic acid, ethyl ester, 9,12-octadecadienoic acid, phosphonic acid, and dioctadecyl ester). The derivatives of phenyl oleic acid are known to inhibit the growth of MCF-7 and HT-29 cancer cells, with IC_50_ values of 48 ppm, whereas n-butyl oleic acid derivatives inhibited the growth of these cells with IC_50_ values of 82 ppm and 77 ppm, respectively [37]. The anticancer activity of ω-6 polyunsaturated fatty acids is known [38]. Compounds isolated from *Cladophora fracta*, such as oleic acid, palmitic acid, gamma-linolenic acid, and linoleic acid, are reported to have strong antiproliferative activity [39]. Anticancer properties are also found in palmitic acid, (Z)-9-octadecenoic acid, and octadecenoic acid isolated from *Protaetia brevitarsis* larvae [40].

Terpenoid compounds were also detected in anticancer isolates from *X. testudinaria* sponge; namely, trans phytol, 1-tricosene, and 2-hexadecen-1-ol, 3,7,11,15-tetramethyl-. These results were in accordance with previous publications, which reported that phytol and diterpene alcohol compounds had anticancer activities in MCF-7 and PC-3 cells, with IC_50_ values of 8.79±0.41 μM and 77.85±1.93 μM, respectively [41]. Other phytol and diterpenes were isolated from *Justicia gendarussa* Burm. f. used as an anti-inflammatory, with histamine release (26.92%), serotine and bradykinin (49.90%), and prostaglandin (68.03%) as compared with the standard (Diclofenac 5 mg/kg) [42]. Phytol also shows anti-angiogenic activity and induces apoptosis in A549 cells by depolarizing the mitochondrial membrane [43,44].

Hydrocarbons (4,8,12,16-tetramethylheptadecan-4-olide; 1-docosene; heneicosane; n-heneicosane) were detected in the anticancer isolate of *X. testudinaria* sponge. Pyrenyl ether, which is a polycyclic aromatic compound with a good cytotoxic effect against cisplatin-induced colon cancer cells (HT-29) and HeLa cancer cells [45]. Essential oil from Gannan navel orange peel, which contains linalool, 3-carene, α-terpineol, decanal, citral, D-limonene, and α-pinene, is reported to have anticancer activity against Hela cells [46]. The sterol fraction of *Taonia atomaria* can inhibit cancer cells: HePG2, A549, HCT116, and MCF7 [47]. The antitumor activity of saturated aliphatic hydrocarbons in *Pyrostegia venusta*, namely, octasane and triacontane compounds from the heptane extract, has also been reported [48].

## Conclusion

The anticancer activity of *X. testudinaria* sponge toxic isolates was positively correlated with the inhibition of HeLa cell growth, with an IC_50_ of 2.273 ppm. In total, 21 compounds were identified in toxic isolate; three groups of compounds were identified, namely, fatty acids and esters, terpenoids, and hydrocarbons.

## Authors’ Contributions

MDS designed and conducted the experiment, acquisition of data, and drafting of the manuscript. WSR conducted the experiment and collected the data. NS designed the experiment, drafting of the manuscript. KKA analyzed the data and drafting the manuscript. All authors read and approved the final manuscript.
